# Relationship Between Psychological Empowerment and the Retention Intention of Kindergarten Teachers: A Chain Intermediary Effect Analysis

**DOI:** 10.3389/fpsyg.2021.601992

**Published:** 2021-02-12

**Authors:** Lina Ma, Fusheng Zhou, Haidan Liu

**Affiliations:** ^1^Faculty of Education, Ningxia University, Yinchuan, China; ^2^Faculty of Education, Ningxia Preschool Education College, Yinchuan, China; ^3^Faculty of Education, East China Normal University, Shanghai, China

**Keywords:** psychological empowerment, psychological capital, job involvement, retention intention, chinese kindergarten teacher

## Abstract

**Objective:** To investigate the relationship between psychological empowerment, psychological capital, job involvement, and the retention intention of kindergarten teachers in mainland China and the internal mechanism of action.

**Methods:** A total of 554 kindergarten teachers were investigated by scales for psychological empowerment, psychological capital, job involvement, and retention intention.

**Results:** (1) Psychological empowerment was positively correlated with psychological capital and job involvement. Psychological capital was positively correlated with job involvement. Psychological empowerment, psychological capital, and job involvement were significantly and positively correlated with retention intention. (2) Psychological empowerment influences kindergarten teachers' retention intention mainly through three indirect effects: the single intermediary effects of psychological capital and job involvement and the chain intermediary effect of psychological capital → job involvement.

**Conclusion:** Psychological empowerment can not only indirectly predict the retention intention of kindergarten teachers through the single intermediary effects of psychological capital and job involvement, but also indirectly predict the retention intention of kindergarten teachers through the chain intermediary effect of psychological capital and job involvement.

## Introduction

Retention intention usually corresponds to turnover intention. Turnover intention is the intensity of an individual's tendency to leave his or her current post and seek other job opportunities (Jg and Simon, 1958), while the intention to not seek other posts is retention intention (Mowday et al., 1979), which reflects the possibility of an individual's self-perception of continuing to work in the organization (Price and Mueller, 1981). Teachers' retention intention is affected not only by objective factors such as school atmosphere (Ingersoll, 2001), occupational reputation (Wynn et al., 2007), working conditions (Kelly, 2004), and leadership support (Brown and Wynn, 2009), but also by demographic characteristics such as teachers' age, teaching experience, and educational background (Hughes, 2012). In addition, occupational commitment (Ingersoll, 2001), job satisfaction (Shann, 1998; Ouyang and Paprock, 2006), and autonomy (Johnson, 2006) have an important impact on teachers' retention intention. At present, the issue of how to retain teachers has become the focus of global attention. A large number of studies have shown that the retention of teachers in recent years is not good, with the estimated range of loss ranging from 20 to 50% (Ingersoll and Smith, 2003; Latham and Vogt, 2007; Perrachione et al., 2008). Researchers agree that the root of the teacher availability problem is not the insufficient supply of teachers but the inability to retain trained teachers (Hammond, 2006). In the current study, few researchers pay attention to retention intention, while many pay more attention to turnover intention, which is often used as a reverse representation of the stability of the teaching staff. However, the reasons for leaving are not always the same as the reasons for staying (Steel et al., 2002). Even when the reasons for leaving and staying are the same, they have two different psychological mechanisms. Thus, the factors that affect retention intention may not be the converse of the factors that affect turnover intention (Yan, 2017). The two-factor theory proposed by Herzberg et al. (1967) can explain the different mechanisms behind resignation and retention. He proposed two different factors, including hygiene factors and motivating factors. Hygiene factors are related to the external environment of work such as working conditions, salary, welfare policies, etc. The deterioration of these factors will result in employee's dissatisfaction, and further cause employees to leave. However, even if these factors are met, they may not lead to positive attitudes. Motivating factors are related to the job, such as achievement, appreciation, development opportunities, autonomy, etc. These factors can stimulate the motivation of individuals to work. When individuals are truly motivated to engage in work, they can maintain the persistence of their career choices. This phenomenon shows that hygiene factors can prevent employees from leaving only to a certain extent, but they cannot effectively promote employees' long-term retention. Zhao's (2018) research also found that merely increasing salary and remuneration did not significantly promote employees' willingness to stay, while nonmonetary incentives (such as those meeting psychological needs) have a more positive significance in retaining teachers. Therefore, it is very necessary to pay attention to teachers' retention intention and to explore in depth the factors that promote it. In addition, most of the existing research has focused on nurses and knowledge workers, while there is less research on teachers, especially on Chinese kindergarten teachers. To sum up, this study takes kindergarten teachers in China as the research object to explore the relationship and mechanism between psychological empowerment and retention intention.

### Psychological Empowerment and Retention Intention

Thomas and Velthouse (1990) proposed the concept of psychological empowerment, believing that psychological empowerment is a synthesis of the psychological state or cognition experienced by individuals, and put forward the cognitive evaluation model theory of psychological empowerment. This theory reflects the whole cognitive process of psychological empowerment, in which intrinsic motivation is the core element of psychological empowerment, and the degree of empowerment depends on employees' evaluation of work tasks in four aspects (meaning, competence, self-determination, and influence). Meaning is an individual's value judgment of the current task and target according to their own values and standards. Competence is the degree to which an individual feels capable of completing a task. Self-determination is the cognition that an individual can make decisions on their method of working, which reflects the individual's autonomy in work. Impact is the extent to which actions affect the outcome of the work being done. In general, when individuals experience a high level of empowerment, they feel the meaning and competence of their work and gain the feeling of independent decision-making and influence over their work. These positive feelings cause them to recognize the organization more; thus, they are more willing to stay in the organization. According to Klerk (2013), a significant relationship obtains between psychological empowerment and retention. He found that individuals were more likely to stay if they experienced a higher level of empowerment. In addition, some recent studies have also shown that psychological empowerment can positively predict retention intention (Iqbal and Hashmi, 2015; Meng et al., 2015; Sandhya and Sulphey, 2020).

### Psychological Empowerment, Psychological Capital, and Retention Intention

Psychological capital is an internal positive psychological resource of individuals that includes four aspects: confidence, hope, optimism, and resilience (Luthans et al., 2004). At present, the concept of psychological capital has been localized in China, where it specifically refers to a measurable, developable, and positive mentality or mental energy possessed by individuals in the process of dealing with people and doing things in the context of an organization (Sun and Zhang, 2013). First, psychological empowerment can positively predict psychological capital. According to social exchange theory, the premise for employees to contribute to the organization is that the organization has certain incentives for employees, so employees can engage in more positive behaviors through the investment of time and energy (Banard, 1938). Therefore, when an organization takes empowerment as an incentive, employees will have a higher experience of psychological empowerment and believe that they are of higher value to the organization. They will face their work in the organization with an optimistic attitude and be full of hope for their future. At the same time, they will contribute more to the organization. Therefore, psychological empowerment can enhance psychological capital. Furthermore, previous studies have reported that psychological empowerment can have a positive effect on psychological capital (Zhang, 2012; Ou, 2017; Hu et al., 2018; Wang D. et al., 2021). Second, psychological capital can positively predict retention intention. We can use the conservation of resources theory (Salanova et al., 2010) to explain the relationship. According to this theory, the accumulation of resources (including relationships and individual resources) is the key driving force to motivate and maintain individual's behaviors, and psychological capital is essentially an important individual resource that can help individuals have positive work attitudes and behaviors as well as loyalty to the organization, so they are willing to work for a long time. Furthermore, the conservation of resources theory (COR) predicts that initial resource gains lead to future gains, thus forming a revenue spiral. This prediction means that individuals will not only maintain and protect existing resources but also strive to obtain more resources, and having psychological capital means they are more able to access other resources, leading to more positive results. The latest research found that psychological capital can not only reduce work stress and job burnout (Kim and Kweon, 2020) but also positively predict life satisfaction, happiness, retention intention, and better performance (Kim and Yoo, 2018; King and Caleon, 2020; Rodríguez-Cifuentes et al., 2020; Santisi et al., 2020).

### Psychological Empowerment, Job Involvement, and Retention Intention

Job involvement is the degree with which an individual psychologically identifies with their work (Lodahl and Kejnar, 1965) and holds an internal cognition or belief in it (Kanungo, 1982), which will change with changes in work environments or characteristics (Li and Long, 1999). On the one hand, psychological empowerment can positively predict job involvement. Psychological empowerment is a motivational construct that comprises individual cognitions and perceptions that constitute feelings of behavioral and psychological investment in work (Conger and Kanungo, 1988; Spreitzer, 1995). Psychological empowerment plays an important role in the attitude and performance of employees. Empowered employees have more intrinsic motivation, they perceive stronger work significance and self-determination in work, and they have a higher sense of self-efficacy (Thomas and Velthouse, 1990). Demir (2020) found that, when teacher self-efficacy was higher, so was their job involvement. Razak et al.'s (2017) survey of 151 bank managers confirmed a positive correlation between psychological empowerment and job involvement. In China, Jiang and Han (2010) and Chen (2013) also found a positive relationship between psychological empowerment and job involvement. On the other hand, job involvement can positively predict retention intention. Job involvement is an individual's psychological recognition of and devotion to their work. Individuals with high job involvement have a stronger sense of accomplishment and identification with their work (Kanungo, 1982). These feelings are an important prerequisite for individual willingness to remain in the organization. According to Brown (1996), job involvement plays a central role in whether an individual stays in organizational work. Compared with people who have low job involvement, people who have high job involvement are much less likely to intend to resign. In recent years, some studies have also found that job involvement can increase the retention intention of individuals (Curtis and Wright, 2001; Yang, 2018; Li et al., 2019).

### Psychological Capital and Job Involvement

A recent study showed that psychological capital as a personal resource can stimulate individual motivation and thereby promote work engagement (Mao et al., 2019). Previous studies on psychological capital have also shown that it can have a positive impact on work input. For example, self-efficacy and optimism as important dimensions of psychological capital can significantly positively predict work input (Halbesleben, 2010; Wang et al., 2017). Other researchers have found that psychological capital not only has a direct positive predictive effect on work input but also can alleviate the negative impact of work requirements on work input (Sheng et al., 2019). In addition, a large number of studies have found that psychological capital can significantly and positively predict job involvement (Wang and Luo, 2015; Cheng and Gao, 2019; Mao et al., 2019; Wang J. et al., 2020).

### Hypotheses of This Study

In summary, the existing research provides a preliminary understanding of the relationship between psychological empowerment, psychological capital, job involvement, and retention intention. We want to verify whether the relationship between the psychological empowerment and retention intention of kindergarten teachers in China is affected by the intermediary role of psychological capital and job involvement, and whether the chain intermediary composed of psychological capital → job involvement is an important means of psychological empowerment affecting the retention intention of kindergarten teachers in China. Therefore, according to the cognitive evaluation model theory of psychological empowerment, social exchange theory, conservation of resources theory, and recent studies, we propose four research hypotheses.

H1: Psychological empowerment can positively predict the retention intention of Chinese kindergarten teachers.
H2: Psychological empowerment can indirectly predict the retention intention of Chinese kindergarten teachers through the intermediary role of psychological capital.
H3: Psychological empowerment can indirectly predict the retention intention of Chinese kindergarten teachers through the intermediary role of job involvement.
H4: Psychological empowerment can indirectly predict the retention intention of Chinese kindergarten teachers through the chain mediating effect of psychological capital and job involvement.

It is of certain theoretical and practical significance to discuss these issues. In terms of theory, it is helpful to explore the process mechanism of psychological empowerment affecting the retention intention of Chinese kindergarten teachers, which may thereof enrich the relevant theoretical basis. It is of more practical significance to discuss the issue of how to retain kindergarten teachers from the perspective of resignation and retention, which is key to ensuring the stability of kindergarten teachers.

## Materials and Methods

### Participants and Procedure

In this study, a convenience sampling method was adopted to collect data from teachers from 20 kindergartens in Yinchuan, Wuzhong, Guyuan, Zhongwei, and Shizuishan in Ningxia, China, from November 1 to November 15, 2019 using the Questionnaire Star online questionnaire platform. To ensure the effectiveness of the network questionnaire, we first contacted the principals of each kindergarten, who then distributed the scale at a teachers meeting, the teachers answered the questions and submitted the scales. All the participants volunteered to participate and were told that the study was for academic purposes and that participants would receive no compensation of any kind. We issued 596 scales. After eliminating the invalid scales, 554 valid scales were obtained, with an effective recovery rate of 93%. Among the participants, 550 were female (99.28%), and four were male (0.72%). One hundred sixty-eight (30.32%) had been teaching for <1 year, 229 (41.34%) had been teaching for 2–5 years, 84 (15.16%) had been teaching for 6–10 years, and 73 (13.18%) had been teaching for more than 11 years. Ninety-eight respondents (17.69%) had a technical secondary school or high school education, 361 (65.16%) had a junior college education, 93 (16.79%) had a bachelor's degree, and 2 (0.36%) had a master's degree or above. This study was approved by the Ethics Committee of Ningxia University.

### Measures

#### Psychological Empowerment

Due to Chen's 2018 revision of the preschool teachers' psychological empowerment scale, the scale includes four dimensions: meaning, competence, self-determination, and impact. The 22 items use a 5-point Likert-type scoring system ranging from one “completely does not conform” to five “completely in line with.” All items together take their average score; when the score is higher, so is the psychological empowerment level. After research and testing, the Cronbach's alpha coefficient for the scale was 0.95, and the Cronbach's alpha coefficient of the four dimensions ranged from 0.88 to 0.92. The confirmative factor analysis results showed that the fitting indexes of the model were ideal (RMSEA = 0.042, NFI = 0.95, CFI = 0.96, GFI = 0.96) (Jin et al., 2020). In addition, Fu et al. (2020) conducted a scale survey among 395 preschool teachers in China and again verified that the scale had good reliability (Cronbach's alpha coefficient was 0.92). The Cronbach's alpha coefficient of this scale in our study was 0.92. The confirmative factor analysis results showed that the fitting indexes of the model in our study were ideal (RMSEA = 0.05, NFI = 0.99, CFI = 0.97, IFI = 0.97).

#### Psychological Capital

The teacher psychological capital scale revised by Zhang Wen (Zhang, 2010) was adopted, which contains 19 items in the four dimensions of confidence, hope, optimism, and resilience. A 6-point Likert-type scoring system is used, which ranges from one “strongly disagree” to six “strongly agree.” The average score of all items is calculated by adding the scores; when the score is higher, so is the psychological capital level. After research and testing, the total scale and Cronbach's alpha coefficient of the four dimensions ranged from 0.61 to 0.80. The confirmatory factor analysis results showed that the fitting indexes of the model were ideal (RMSEA = 0.06, IFI = 0.88, CFI = 0.88, TLI = 0.84) (Peng et al., 2018). In China, some other studies have tested the Cronbach's alpha coefficient of this scale with results from 0.82 to 0.84 (Liu and Zhou, 2016; Zhou et al., 2019; Qiu and Wang, 2020). The Cronbach's alpha coefficient of this scale in our study was 0.87. The confirmative factor analysis results showed that the fitting indexes of the model in our study were ideal (RMSEA = 0.07, NFI = 0.91, CFI = 0.93, IFI = 0.93).

#### Job Involvement

The job involvement scale developed by Kanungo in 1982 and translated by Zhou and Li (2006) consists of 10 independent items. A 5-point Likert-type scoring system is used, which ranges from one “strongly disagree” to five “strongly agree;” the second and seventh items are reverse scored, and the rest are graded forward. The scores of all the items are added to obtain an average. When the score is higher, so is the level of work involvement. The Cronbach's alpha coefficient of the original scale was 0.87 (Kanungo, 1982). The Cronbach's alpha in the Chinese version is 0.85, and the KMO test coefficient is 0.88 (Zhou and Li, 2006), showing good reliability and validity. In China, some other studies have also tested the Cronbach's alpha coefficient of this scale with results between 0.84 and 0.87 (Hu and Qiu, 2016; Liu and Gu, 2018). The Cronbach's alpha coefficient of this scale in our study was 0.82. The confirmative factor analysis results showed that the fitting indexes of the model in our study were ideal (RMSEA = 0.044, NFI = 0.98, CFI = 0.99, IFI = 0.99).

#### Retention Intention

The kindergarten teacher retention intention scale prepared by Shi (2019) was adopted, which contains eight independent items. A 5-point Likert-type scoring system is used, which ranges from one “strongly disagree” to five “strongly agree.” The average score of all items is calculated by adding the scores; when the score is higher, so is the retention intention. The Cronbach's alpha and KMO coefficients of the original scale were 0.95 and 0.92, respectively (Shi, 2019). Guo and Cai (2021) tested the reliability and validity of the scale, and the results showed that the Cronbach's alpha coefficient was 0.93, and the confirmative factor analysis results showed that the fitting indexes of the model were ideal (RMSEA = 0.046, NFI = 0.996, CFI = 0.997, GFI = 0.994). The Cronbach's alpha coefficient of this scale in our study was 0.91. The confirmative factor analysis results showed that the fitting indexes of the model in our study were ideal (RMSEA = 0.054, NFI = 0.99, CFI = 0.99, IFI = 0.99).

### Data Analysis

To ensure the objectivity and authenticity of the research results, the scale was completed anonymously on the Internet. After the teacher respondents answered the scale and basic information anonymously through the scale link, they submitted the completed scales through the Internet platform. IBM SPSS 22.0 was used for preliminary data processing, descriptive statistics, and the reliability and correlation analyses among the variables. Model 6 in the SPSS macro program (http://www.afhayes.com) was used to analyze the mediating effect of psychological capital and job involvement in the relationship between psychological empowerment and retention intention.

### Assessment of Common Method Variance

To test the common method deviation or systematic measurement error caused by the self-described questionnaire collection of all data, the Harman single factor test was carried out in the study (Harman, 1976), and an exploratory factor analysis was performed on all items consisting of four variables, namely, psychological empowerment, psychological capital, job involvement, and retention intention. The results showed that nine factors had eigenvalues >1. The first factor explained 30.80% of the total variation, which was less than the 40% threshold criterion proposed by Podsakoff et al. (2003). This result does not eliminate the possibility of common method variance; however, it suggests that common method variance is unlikely to confound the interpretations of the data analysis results.

## Results

### Descriptive Statistics and the Correlation Between the Main Variables

Table 1 shows the general means, standard deviations, and correlation coefficients of psychological empowerment, psychological capital, job involvement, and retention intention. The results suggest that all the variables are significantly correlated with each other. Psychological empowerment is positively correlated with psychological capital and job involvement (*r* = 0.50, *p* < 0.01; *r* = 0.47, *p* < 0.01). Psychological capital is positively correlated with job involvement (*r* = 0.58, *p* < 0.01). Psychological empowerment, psychological capital, and job involvement are significantly positively correlated with retention intention (*r* = 0.44, *p* < 0.01; *r* = 0.56, *p* < 0.01; *r* = 0.64, *p* < 0.01).

**Table 1:** Means, standard deviations, and correlations of the major study variables.

**Variable**	**M**	**SD**	**1**	**2**	**3**	**4**
1. Psychological empowerment	3.89	0.61	1.00			
2. Psychological capital	4.52	0.60	0.50^**^	1.00		
3. Job involvement	3.58	0.57	0.47^**^	0.58^**^	1.00	
4. Retention intention	3.90	0.68	0.44^**^	0.56^**^	0.64^**^	1.00

*N = 554*.

**p < 0.01; All tests were two-tailed. This table shows the general means, standard deviations, and correlations of the four major variables. **indicates a significant correlation between the variables, which obtains between all the variables.

### Chain Mediation Model Analysis

Psychological empowerment, psychological capital, job involvement, and retention intention are significantly correlated, which meets the statistical requirements for the further mediating effect analysis of psychological capital and job involvement (Wen and Ye, 2014). After controlling for the teaching experience and educational background, Model 6 in the SPSS macro program (http://www.afhayes.com) compiled by Hayes (2012) was used to analyze the mediating effect of psychological capital and job involvement in the relationship between psychological empowerment and retention intention.

Table 2 shows the regression analysis results of the relationship between psychological empowerment and retention intention, in which teaching experience and educational background are the control variables. The results show that psychological empowerment has a significant positive predictive effect on retention intention (B = 0.419, *p* < 0.001). Hypothesis 1 has been tested. When psychological capital and job involvement are included in the regression equation, psychological empowerment significantly predicts psychological capital (*B* = 0.500, *p* < 0.001) and job involvement (*B* = 0.226, *p* < 0.001). Psychological capital significantly predicts job involvement (*B* = 0.464, *p* < 0.001) and retention intention (*B* = 0.249, *p* < 0.001). In addition, job involvement is a significant positive predictor of retention intention (*B* = 0.444, *p* < 0.001). At this point, the direct effect value of psychological empowerment on retention intention is significantly reduced (*B* = 0.091, *p* < 0.05). These results indicate that psychological capital, job involvement and the chain mediating effect of psychological capital → job involvement are significant among the influences of psychological empowerment on retention intention. Hypotheses 2–4 are confirmed.

**Table 2:** Regression analysis of the relationship between psychological empowerment and retention intention.

**Regression equation**		**Fitting index**			**Significance**	
**Result variable**	**Predictor variable**	**R**	***R*^**2**^**	**F**	**B**	**t**
Retention intention		0.450	0.202	46.485^***^		
	Teaching experience				0.073	2.785^**^
	Educational background				−0.068	−1.188
	Psychological empowerment				0.419	10.810^***^
Psychological capital		0.504	0.254	62.300^***^		
	Teaching experience				0.011	0.414
	Educational background				−0.001	−0.023
	Psychological empowerment				0.500	13.333^***^
Job involvement		0.621	0.385	85.965^***^		
	Teaching experience				0.037	1.599
	Educational background				−0.011	−0.228
	Psychological capital				0.464	11.967^***^
	Psychological empowerment				0.226	5.775^***^
Retention intention		0.691	0.478	100.237^***^		
	Teaching experience				0.052	2.436^*^
	Educational background				−0.062	−1.343
	Job involvement				0.444	11.266^***^
	Psychological capital				0.249	6.213^***^
	Psychological empowerment				0.091	2.451^*^

*N = 554*.

* *p < 0.05*,

** *p < 0.01*,

*** *p < 0.001. All variables in the model have been standardized. Teaching experience and educational background are the control variables, psychological empowerment is an independent variable, and retention intention is a result variable. This table presents the results of the multiple hierarchical regression analysis of psychological empowerment, psychological capital, job involvement, and retention intention*.

**Figure 1:**
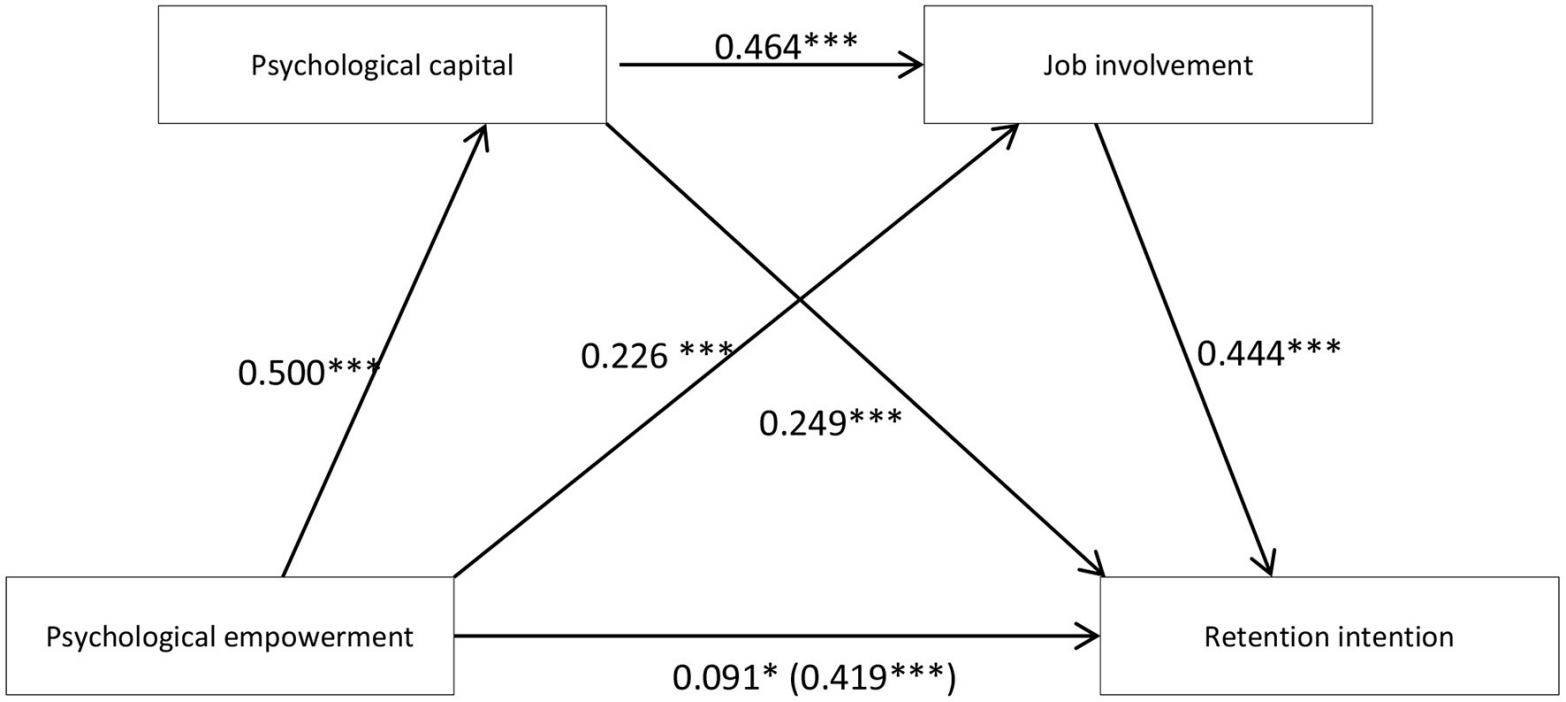
The chain mediation model. The chain mediation model shows the effects of psychological empowerment, psychological capital, and job involvement on the retention intention. *N* = 554. The effect of psychological empowerment is shown in parentheses. After controlling for teaching experience and educational background in the PROCESS program of SPSS, the regression coefficient was obtained. ***p < 0.001,*p < 0.05.

Table 3 shows the mediating effect value of psychological capital and job involvement between psychological empowerment and retention intention. Figure 1 is a chain mediating model between psychological empowerment and job involvement. Table 3 and Figure 1 show that psychological capital and job involvement play a significant mediating role between psychological empowerment and retention intention, and the total standardized mediating effect value was 0.328. Specifically, the mediating effect is composed of three indirect effects: path 1—psychological empowerment → psychological capital → retention intention (0.124), path 2—psychological empowerment → job involvement → retention intention (0.101), and path 3—psychological empowerment → psychological capital → job involvement → retention intention (0.102). The ratios of the three indirect effects to the total effect are 29.83, 23.87, and 24.58% for paths 1, 2, and 3, respectively, and the 95% confidence interval of the above indirect effects does not contain the zero value, indicating that all three indirect effects reach a significant level. Hypotheses 2–4 are thus confirmed again. Comparison 1 shows that the bootstrap 95% confidence interval for the difference between indirect effects 1 and 2 contains a 0 value, indicating that there is no significant difference between them. Using the same comparison approach, no significant difference is found between indirect effects 1 and 2 or between indirect effects 2 and 3. These results indicate that psychological empowerment can indirectly predict retention intention not only through the single mediating effect of psychological capital and job involvement but also through the chain mediating effect of psychological capital and job involvement. The single mediating effect of psychological capital accounted for the highest ratio of the total effect (29.83%).

**Table 3:** Psychological capital and job involvement in the mediation effect analysis.

	**Indirect effects**	**Boot SE**	**Boot LLCI**	**Boot ULCI**	**Relative mediation effect**
Total indirect effect	0.328	0.034	0.266	0.397	78.28%
Indirect effect 1	0.125	0.027	0.076	0.183	29.83%
Indirect effect 2	0.100	0.023	0.058	0.150	23.87%
Indirect effect 3	0.103	0.016	0.074	0.134	24.58%
Compare 1	0.024	0.040	−0.058	0.100	
Compare 2	0.022	0.031	−0.040	0.084	
Compare 3	−0.002	0.027	−0.056	0.050	

*The direct and indirect effects of psychological empowerment on retention intention are shown in this table. Boot SE, Boot LLCI, and Boot ULCI refer, respectively, to the standard error and the lower and upper limits of the 95% confidence interval of the indirect effects estimated by the percentile bootstrap method with deviation correction. Indirect effect 1: psychological empowerment → psychological capital → retention intention; indirect effect 2: psychological empowerment → work involvement → retention intention; indirect effect 3: psychological empowerment → psychological capital → job involvement → retention intention*.

## Discussion

The results of this study show that the mediating effects of psychological capital and job involvement may contribute to understanding the relationship between psychological empowerment and retention intention in a sample of Chinese kindergarten teachers. First, consistent with a prior study (Meng et al., 2015), we found that psychological empowerment can significantly positively predict the retention intention of kindergarten teachers in China; that is, when the psychological empowerment level is higher, the retention intention is stronger. This result means that psychological empowerment in the Chinese context is an important incentive for kindergarten teachers' retention intention. The experience of empowerment enhances their perception of their own value and the meaning of their work, thus improving their sense of competence and autonomy in completing their work, which is of positive significance in encouraging them to stay in kindergartens for a long time.

Although previous studies have confirmed that psychological empowerment can affect retention intention, how does psychological empowerment affect retention intention? The internal process mechanism is not clear enough. Previous studies have found that psychological empowerment can improve employee satisfaction, thus promoting employee retention (Elnaga and Imran, 2014; Fernandez and Moldogaziev, 2015). Our study further examined the relationship between psychological empowerment and retention intention from different perspectives. For the first time, we found that psychological capital is a mediating variable by which psychological empowerment affects retention intention. This finding means that psychological empowerment can promote retention intention by enhancing psychological capital. China has a typical cultural characteristic of high power distance, and kindergartens often adopt a highly centralized teaching management mode (Wang et al., 2015). In such a context, the empowerment experience is extremely valuable. Once kindergarten teachers are empowered, they are liberated from the high-control mode; thus, they feel that they have influence and autonomy over their work. In exchange, they will be full of trust and hope for kindergarten, and they will treat their work more positively and confidently, which plays an important role in enhancing their psychological capital. This finding further explains the viewpoint of social exchange theory (Banard, 1938). In addition, some studies have found that psychological capital is a protective factor against pressure and challenges (Wang et al., 2014). Kindergarten teachers in China are generally faced with heavy tasks. They shoulder up high expectations from parents, principals and social groups, so they need to do a better job in child care and education. Furthermore, they also need to face various emergencies while ensuring the safety of children (Wang et al., 2014). Therefore, they are under great physical and psychological pressures and are prone to depression, anxiety, burnout and other negative psychological states. Under this kind of high working pressure, psychological capital is particularly important. Kindergarten teachers with high psychological capital have more personal resources, which are conducive to helping them in a high-stress working environment to treat pressures and difficulties as challenges rather than threats so they can adopt a positive and optimistic coping style, which enhances their retention intention. This further explains the theory of conservation of resources (Salanova et al., 2010) and is consistent with previous research results (Kim and Yoo, 2018).

In the mechanism of psychological empowerment affecting the retention intention of kindergarten teachers in China, we also found that job involvement is an important mediating variable. Previous studies have proven that job involvement is an intermediary of psychological empowerment affecting turnover intention, that is, people with psychological empowerment are more engaged, and these people reflect a lower turnover intention (Bhatnagar, 2012). Our study verifies the role of job involvement in psychological empowerment and retention intention from a positive perspective, which indicates that psychological empowerment can enhance retention intention by promoting job involvement. First, we found that psychological empowerment promotes job involvement. Previous studies have shown that psychological empowerment is an important incentive resource and can enhance employees' job involvement (Ugwu et al., 2014; Razak et al., 2017), our study confirms this relationship. The nature of kindergarten teachers' work is different from that of primary and secondary school teachers. The educational object they face is immature children, so they need more of a sense of responsibility. At the same time, the curricula and activities of kindergarten are flexible and generative. Therefore, kindergarten teachers need more autonomy in their daily work. Psychological empowerment can well meet the requirements of kindergarten teachers, which allows them to perceive the value of their work and willing to have more patience and a greater sense of responsibility. At the same time, autonomy can ensure kindergarten teachers work efficiently and experience a sense of accomplishment. The high sense of accomplishment will cause them to be immersed in their work. In this state, they are enthusiastic about their work and willing to be energetic in achieving their goals (Consiglio et al., 2016), further enhancing their job involvement. At the same time, job involvement further promotes retention intention. The increase of job involvement means the increase of the psychological identification degree of kindergarten teachers, which inspires them to love the occupation of preschool teachers more and produces a strong sense of belonging and loyalty, which plays an important role in improving their retention intention.

Finally, we found for the first time that the chain intermediary constituted of psychological capital → job involvement is also an important way psychological empowerment affects the retention intention of kindergarten teachers in China. This finding means that psychological empowerment can enhance the psychological capital of kindergarten teachers, and psychological capital can promote their job involvement to further promote the retention intention. This finding also indicates that the process of psychological empowerment's influence on retention intention is relatively complex. Although our study has found the separate mediating effects of psychological capital and job involvement, as well as the chain mediating effect they form, they all produce partial mediating effects. Therefore, they cannot fully explain the relationship between psychological empowerment and retention intention. In fact, other factors may be at work, which is worth further study. Furthermore, we find that, among all the paths in which psychological empowerment affects the retention intention of kindergarten teachers in China, the path of psychological empowerment → psychological capital → retention intention has the highest indirect effect value, accounting for 29.83% of the total effect. This result shows that psychological capital is very important for kindergarten teachers who experience great pressure and carry a large burden in China, and it is an important “incentive factor” in promoting the retention intention of kindergarten teachers. Therefore, in exploring how to retain kindergarten teachers in China, we should pay more attention to the construction of teachers' internal psychological resources.

### Conclusion, Implications, and Limitations of the Study

In conclusion, the purpose of our study was to explore the relationship and mechanism between the psychological empowerment and retention intention of kindergarten teachers in China. We constructed a chain intermediary model and found that psychological empowerment can not only directly predict retention intention but also indirectly predict retention intention through the separate mediating effects of psychological capital and job involvement. Meanwhile, it can also indirectly predict retention intention through the chain mediating effect of psychological capital and job involvement.

This study can provide insights for effectively improving the retention intention of kindergarten teachers. First, psychological empowerment can directly predict the retention intention of kindergarten teachers. Therefore, attention should be paid to the empowerment experience of kindergarten teachers, which should not only improve their sense of work meaning and impact through empowerment behavior but also enhance their sense of self-determination and competence through personalized care to improve their retention intention. Second, psychological empowerment can affect the retention intention of kindergarten teachers through psychological capital, job involvement and the chain intermediary between these two factors, which indicates that psychological capital and job involvement are key factors affecting the retention intention of kindergarten teachers. On the one hand, attention should be paid to the construction of kindergarten teachers' psychological resources; Luthans' psychological capital intervention (PCI) model (Luthans et al., 2005) can be used as a reference for improving kindergarten teachers' psychological capital based on the four dimensions of confidence, hope, optimism, and resilience. On the other hand, attention should be paid to kindergarten teachers' cognition and attitude toward their work. Only by helping kindergarten teachers have positive cognition and belief toward their own work value can their job involvement level be promoted.

This study also has some shortcomings. First, due to space and time limitations, this study adopted a cross-sectional study design. Although previous studies have provided a foundation for this type of study, it is still difficult to draw an exact causal relationship using this approach. In future studies, longitudinal or experimental approaches could be used to further investigate the causal relationship of various variables. Second, the results of this study cannot be applied to all kindergarten teachers. The object of this study is kindergarten teachers in China, 99% of whom are female; thus, the sex ratio is not balanced. In future studies, the sex ratio of the participants should be considered, and the retention willingness of kindergarten teachers in different countries should be studied for comparisons. Third, since this study adopted a self-report questionnaire approach, it is difficult to exclude the possibility that the respondents' answers are conservative or exaggerated compared with the actual situation; therefore, future studies should try to collect data from multiple information sources.

## Data Availability Statement

The raw data supporting the conclusions of this article will be made available by the authors, without undue reservation.

## Ethics Statement

The studies involving human participants were reviewed and approved by Ethics Committee of Ningxia University. The patients/participants provided their written informed consent to participate in this study.

## Author Contributions

LM: propose hypotheses, collect data, and complete paper writing. HL: analysis data and translation. FZ: revise and perfect the thesis. All authors contributed to the article and approved the submitted version.

## Conflict of Interest

The authors declare that the research was conducted in the absence of any commercial or financial relationships that could be construed as a potential conflict of interest.
